# Exercise and nutrition interventions for renal cachexia

**DOI:** 10.1097/MCO.0000000000001022

**Published:** 2024-02-21

**Authors:** Adrian Slee, Joanne Reid

**Affiliations:** a Division of Medicine, Faculty of Medical Sciences, University College London (UCL); b School of Nursing and Midwifery, Queen's University Belfast (QUB), Belfast, Northern Ireland, UK

**Keywords:** cachexia, chronic kidney disease, exercise, omega-3, oral nutritional supplementation, resistance training

## Abstract

**Purpose of review:**

Renal cachexia is a deleterious condition characterized by weight loss, muscle wasting and loss of physical function, quality of life, and increased mortality. Multimodal treatment strategies utilizing exercise and nutrition interventions have been recently suggested although the evidence base is still in its infancy. This paper aimed to review the current literature surrounding the use of exercise and nutrition for renal cachexia.

**Main findings:**

Evidence from systematic reviews and narrative reviews indicates that resistance training (RT) is proven to have beneficial effects on improving muscle strength and in some cases physical function, although effects on muscle mass are mixed and inconclusive. Further, combined RT and aerobic training (AT) may have also beneficial effects on overall functional capacity and there appears to be no superior mode of protocol (inter/intra-dialysis vs. home-based). For nutrition, there is new data from systematic review of studies indicating that oral nutritional supplementation (ONS) may have beneficial effects on nutritional status (e.g. body mass index, plasma albumin and handgrip strength). Omega-3 fatty acids have been shown to have anti-inflammatory effects in haemodialysis patients from two recent systematic reviews, and evidence from other populations groups indicate they may be beneficial for improving muscle mass and strength.

**Summary:**

Evidence is accumulating for individual exercise and nutrition components but specific multimodal treatment studies in renal cachexia need to be urgently undertaken.

## INTRODUCTION

A number of overlapping nutrition-related syndromes are prevalent in chronic kidney disease (CKD) patients, such as disease-related malnutrition, cachexia, protein-energy wasting (PEW), sarcopenia and frailty [2–5] Cachexia has been defined as “a complex metabolic syndrome associated with underlying illness and characterized by loss of muscle with or without loss of fat mass”, and linked to reduced quality of life (QoL), increased morbidity, disability and mortality [6]. Cachexia is closely related to PEW and may be considered as a more progressive and advanced degree of wasting [3]. Typically, key features of cachexia include: weight loss (and low body mass index, BMI), anorexia, inflammation, insulin resistance and increased muscle protein breakdown [6]. The net result leads to wasting and a specifically a loss of skeletal muscle mass, strength, functional capacity, with symptoms of weakness and fatigue being common. In CKD dialysis patients, there are complex interacting factors that can impact, leading to the development of PEW/cachexia. For example, the dialysis process [e.g. haemodialysis, (HD)] itself is catabolic with amino acids lost in the dialysate, and dialysis may activate inflammatory pathways [7]. The CKD disease process itself and presence of other comorbidities (e.g. cardiovascular disease, heart failure or type 2 diabetes) may also heighten proinflammatory cytokine release [e.g. interleukin (IL)-1, IL-6 and tumour necrosis factor (TNF)α], known to have direct effects on activation of the acute phase response and skeletal muscle proteolysis (e.g. upregulation of gene expression of components of the ubiquitin-proteasome system), inhibition of muscle protein synthesis, and may cause a hypothalamic anorectic response, reducing food intake [8]. In CKD, there is also apparent anabolic resistance or impaired signalling of key anabolic pathways such as insulin and insulin-like growth factor-1 (IGF-1) due to factors such as inflammation, acidosis, uremic toxins and raised glucocorticoids [9]. Coupled with many other common factors in this patient group such as ageing, physical inactivity, dietary restrictions, vitamin D and sex hormone deficiencies (e.g. testosterone), skeletal muscle wasting, sarcopenia and associated symptoms such as weakness and fatigue are very common [2]. 

**Box 1:**
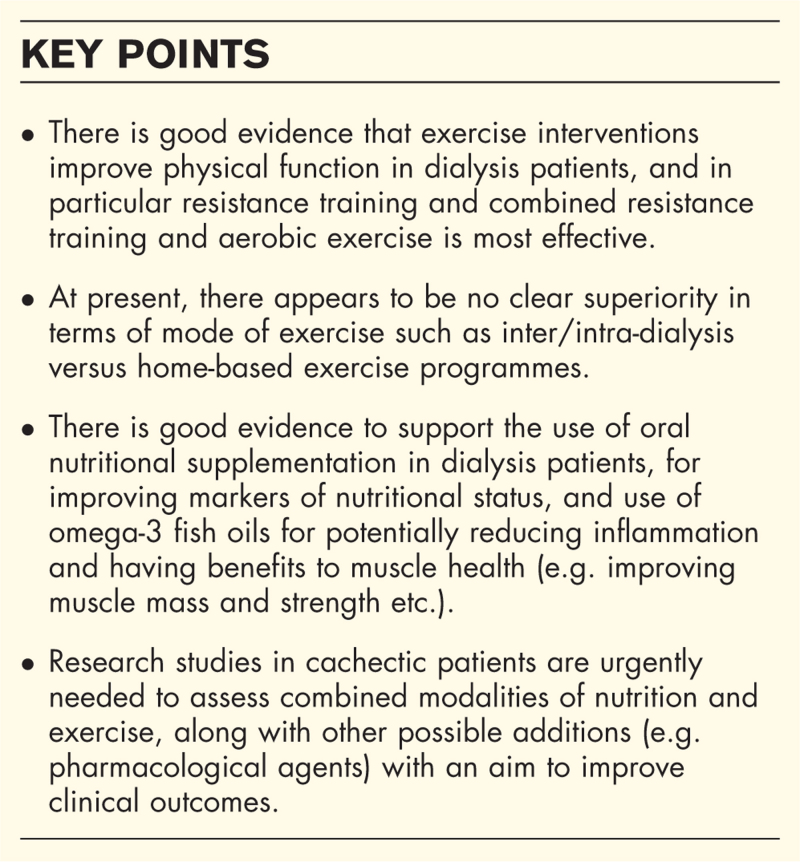
no caption available

Our group has studied cachexia prevalence in a cohort of HD patients from Northern Ireland, United Kingdom (UK), and using the Evans *et al.*[6] criteria and found that at baseline 16% were cachectic (17 of 106 patients) [10]. Note there was no significant difference in number of males/females, or Charlson comorbidity index (CCI) or other indicators of disease burden between cachectic and noncachetic patients. It was also found that in all patients there was worsening of symptoms, e.g. FACIT scores indicating fatigue significantly worsened in cachectic patients over a 12 month period, as did hand grip strength, indicating a physical decline. In fact, one overwhelming feature of the study was that all HD patients (± cachexia) exhibited very high levels of muscle weakness/dynapenia, regardless of which recommended published cut off points were used for handgrip strength (HGS), with 50–80% patients having significant muscle weakness [11]. This may be due to a combination of factors such as physical inactivity/deconditioning, disease burden, neuromuscular alterations, changes in muscle fibre type; and other prominent factors, such as fat infiltration in skeletal muscle as recently discussed in a review by [12].

Related syndromes such as sarcopenia have been shown to be linked to poor clinical outcomes in CKD patients. A recent systematic review and meta-analysis of studies (50 studies with 72 347 patients) showed that low muscle strength, mass and physical performance were associated with higher mortality in a range of CKD patients [13]. Furthermore, another recent systematic review and meta-analysis of studies specifically in dialysis patients (30 studies and 6162 patients) showed that sarcopenia prevalence is high and linked to higher mortality [14].

**FIGURE 1:**
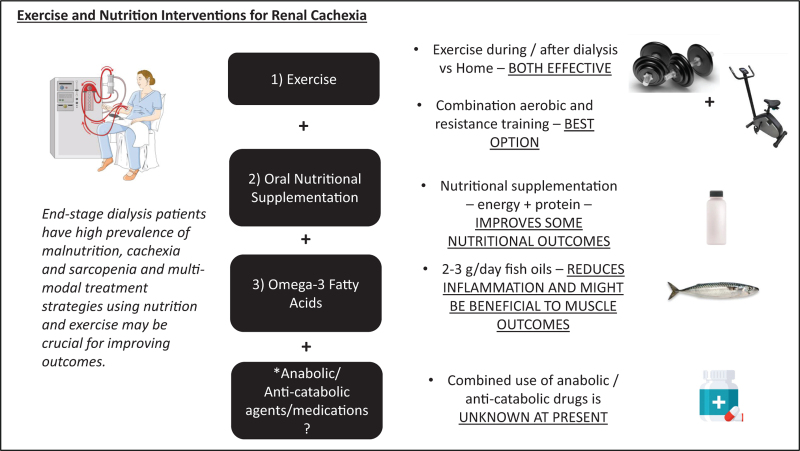
Figure to show the key aspects of discussion within the paper and the current evidence, including the use of exercise modalities, oral nutritional supplementation and omega-3 fatty acids. There is also an indication that an addition of an anabolic/anticatabolic agent might be beneficial in a multimodal context of intervention but needs to be tested and proven in future studies.

Treatment strategies to combat cachexia and related muscle wasting syndromes are essential for improving patient outcomes, and multimodal combination therapies have been suggested and have been trialled in disease conditions, such as cancer and chronic obstructive pulmonary disease (COPD), as outlined in a critical review [15]. In particular, given the evidence of high prevalence of muscle weakness/dynapenia/sarcopenia in CKD patients, exercise and specifically muscle strengthening/resistance exercises might be most beneficial alongside nutrition and other modalities such as pharmacological agents. Our group recently published a theoretical framework, developed collaboratively with key stakeholders (including patients, carers and healthcare professionals), relating to a multimodal, integrative, exercise, anti-inflammatory and dietary counselling intervention (named MMIEAD) for renal cachexia. Importantly, this paper presents a theory of change which details the: causal pathways for renal cachexia; multimodal intervention components; anticipated outcomes, and evaluation methods (1). This review aims to briefly highlight some current trends in the literature around this topic, specifically on exercise and nutrition strategies to combat renal cachexia (Fig. 1).

## EXERCISE STRATEGIES FOR RENAL CACHEXIA

### Resistance training

As previously indicated it is considered that resistance training (RT)/strengthening exercises should be a key feature of any multimodal cachexia intervention to combat muscle weakness and wasting. Specifically relating to RT and strength training in CKD patients, Molsted *et al.*[16] published a systematic review analysing eight studies in HD patients (*n* = 290 total). Four of these studies were intra-dialysis and four inter-dialysis interventions using RT, and they found there was a generally consistent effect of RT improving muscle strength and some evidence of improved physical function, but however, inconsistent effects on increasing muscle mass. Our group conducted a narrative review assessing RT studies, some of which also included specific nutrition interventions (such as high protein ONS and vitamin D) and their impact upon sarcopenia outcomes [17]. This assessment included 14 resistance training studies and a further five which included nutritional interventions, all published between 2010 and 2020. Our analysis indicated similar results with consistent effects on muscle strength, and some on performance, but also with inconsistent effects on muscle mass. Of the high protein ONS nutritional studies [4], we found they also had a beneficial effect but no consistency with improving muscle mass, and this might relate to dose used. We noted that key factors relating to exercise participation and adherence, longer term intervention durations, progressive loading and higher intensities were potentially favourable in promoting significant gains.

### Evaluating different training modalities

When considering feasibility and superiority of exercise programmes one key aspect to consider is whether an intra-/interdialytic (either during or around the time of dialysis) exercise programme is better than a home-based exercise programme, or other exercise modalities. This is something that has been recently assessed in a network meta-analysis of randomized controlled trials in HD patients (78 studies, *n* = 3326) [18^▪▪^]. This has also been recently discussed in an Editorial by Zoccali *et al.*[19]. Currently, evidence seems to suggest that either of these modalities (intra-/interdialytic vs. home-based) are beneficial with neither showing superiority in terms of outcomes. In the meta-analysis by Ferrari *et al.*[20,21] interestingly and of high importance they found that combined RT and aerobic training (AT) was more beneficial, and that interventions >12 weeks and those with moderate-vigorous intensity yielded better effects on improvements in functional capacity. These results appear logical as a longer period of time may be necessary for significant physiological benefits to manifest. In addition, in population studies higher moderate-vigorous physical activity levels have been shown to correlate with muscle mass and strength and be protective of sarcopenia risk.

Most recent examples of relevant exercise studies are as follows: a large multicentre randomized controlled trial in HD patients (1211 recruited and 446 patients in intervention group included in analysis, and 471 controls) was performed by a German group (the DiaTT trial) investigating the effects of a 12-month intradialytic RT + AT intervention [22]. They showed that the intervention had a significant positive effect on measures of physical function (60 s sit-to-stand test and 6 min walk test) along with some aspects of quality of life (physical health and vitality sub-scores of 36-item Short Form Health Survey) [23^▪▪^]. This was a well powered study with high patient numbers, and long duration of intervention (12 months), and was discussed in the Editorial by Zoccali *et al.*[19] comparing this study to other known trials in HD patients. A further recent randomized controlled trial using a 6-month combined intradialytic RT + AT intervention was performed by an Iranian study group in 74 HD patients [24]. The primary outcome was survival and the study showed that the combined RT+AT improved survival after 12 months. Secondary outcomes that improved, relevant to cachexia, included physical function [6-min walking test (6MWT)], nutritional status (albumin and Geriatric Nutritional Risk Index) and haemoglobin (all *P* < 0.001). Note here that it is probable that the dose and duration of intervention was adequate to promote a significant beneficial response (i.e. 60 min × 3 times per week for 6 months). Another small study in male HD patients from Greece trialled a 4-month intradialytic RT + AT programme [25]. This study was randomized with a control group (*n* = 15 intervention and *n* = 14 controls). They found that the exercise group had significant increases in HGS (*P* < 0.05), 6MWT (*P* < 0.05) lean mass (*P* = 0.01) and bioelectrical impedance assessment (BIA) phase angle (*P* = 0.04). The last intradialytic exercise study to report is a recent pilot trial from a Mexican group that compared intradialytic ONS (two cans of ONS consumed, one during dialysis, and one after, and each can contained 434 kcal and 19.2 g protein), with ONS + exercise (RT + AT) [26]. The study intervention lasted for 6 months and led to improvements in HGS and physical function in both groups, with a greater effect size in the ONS + exercise group. However, interesting to note that both muscle mass and muscle quality, assessed by gold standard computed tomography scanning, did not alter. Similar study issues are discussed and should be considered such as low study patient recruitment (*n* = 14 patients in the ONS group and *n* = 10 in the ONS + exercise), and overall power of the intervention to counteract catabolism. For example, as discussed in their paper, whether the exercise intensity utilized was sufficient to stimulate hypertrophy.

A recent example of a home-based programme is the recent UK Kidney-BEAM study [27]. This trialled the use of a 12-week multicentre, randomized remote digital health platform encouraging kidney disease patients (*n* = 340 CKD patients, with 20% and 22% at stages 4 and 5, respectively) to engage in physical activity (RT + AT) using online resources – live and prerecorded sessions alongside education and motivation support delivered by specialist kidney diseases physical therapists [27]. Those patients in the intervention group were found to have significantly improved measures of quality of life (KDQoL-SF.13) and 60 s sit to stand test and some improvements in symptoms of fatigue.

In summary, it appears that muscle strengthening RT and combined RT + AT interventions have a good evidence base supporting there use in a potential cachexia multimodal treatment programme. In terms of specific mode of intervention, this is something that should be individualized to the patient and based upon feasibility, practicality and costs which needs to be considered and assessed [19].

## NUTRITIONAL INTERVENTIONS FOR RENAL CACHEXIA

### Nutritional guidelines and nutritional counselling

As discussed above, exercise tends to have beneficial effects on markers of functional status (e.g. muscle strength and physical function), but this is not evident or consistent for markers of muscle mass. For CKD patients with cachexia who may be in a negative energy and protein balance (with hypermetabolism and hypercatabolism), it is critical that energy and protein requirements are met in an effort to promote a positive energy balance, counteract the catabolism relating to disease factors and to support any exercise-induced anabolism (e.g. amino acid stimulated muscle protein synthesis postexercise).

Hence, the key focus of all nutritional interventions with CKD patients should be on promoting adequate energy and protein intake through individualized nutritional counselling. The current Kidney Disease Outcomes Quality Initiative (KDOQI) guidelines state that energy intake should be 25–35 kcal/kg/day [28], but note that in cachectic patients with inflammation and hypermetabolism the upper end might be suggested (i.e. 30–35 kcal/kg/day) and should be personalized on individual patient assessment (patient needs, weight loss, energy expenditure, inflammation, body composition (lean mass/fat mass ratio)). Current KDOQI recommendations for protein intake are 1.0–1.2 g/kg/day for dialysis patients [28]. The protein intake recommendations have also been discussed in some detail in a recent specialized review, agreeing with the heightened recommendations for those patients on dialysis, and potentially higher levels may be necessary for those with severe muscle wasting, which may come from additional ONS if patients find it hard to consume additional food [29^▪^]. In terms of sources of protein, there is has been a shift towards an interest in plant-based protein sources due to their effects on acid-base balance [29^▪^,^30^]. However, they tend to have less anabolic potential due to their amino acid profile (e.g. reduced essential amino acids such as lysine and methionine) and hence for CKD patients on HD, greater focus on higher quality protein sources might be considered a better approach until new research is made available, or combining different types of plant proteins to maximize the amino acid pattern [29^▪^,^30^].

### Oral nutritional supplementation

If nutritional counselling by itself is not sufficient in improving nutritional status, then ONS can be suggested and prescribed. ONS has already been mentioned in this paper with the combined ONS and exercise study [26].

A recent Brazilian study trialled use of a tailored ONS supplement plan in older (>65 years of age) HD patients [31]. Nutritional counselling was given to both groups of patients (±Supplement). They found that after one year those patients +Supplement had significantly preserved skeletal muscle mass index values (SMMI), measured by multifrequency BIA. They also showed that those patients who died (12-month mortality as secondary outcome) had significantly lower SMMI values (*P* = 0.017), and higher C-reactive protein (CRP) (*P* = 0.046), as a marker of inflammation. Note that the +Supplement group at baseline was older and had worse nutritional status, with significantly lower body weight, BMI, calf circumference (all *P* < 0.001), and SMMI values (*P* = 0.049).

Furthermore, a recent systematic review and meta-analysis was performed investigating the effects of ONS on nutritional status and inflammation in dialysis patients [32^▪▪^]. The study included 22 randomized controlled trials (RCTs) with 1185 patients included for the meta-analysis. Overall, they found that the ONS intervention groups had significantly improved markers of nutritional status, including BMI (*P* = 0.005), plasma albumin (*P* < 0.0001) and HGS (*P* = 0.034), however, there were no significant changes in lean body mass. Other markers were analysed such as BIA phase angle and plasma CRP, but these did not significantly alter, and this is perhaps to be expected as only three studies of each were assessed.

### Omega-3 polyunsaturated fatty acids

A hallmark of cachexia is disease-related inflammation, and hence a key feature of any multimodal strategy to combat cachexia should consider use of anti-inflammatory agents. Omega-3 polyunsaturated fatty acids (n-3 PUFAs) are essential fatty acids found in the diet (e.g. in marine food, oily fish, and precursor alpha linolenic acid in plant-based foods such as walnuts and flaxseed) and have been shown to have potent anti-inflammatory effects in a range of studies. Principally, the two main fatty acids eicosapentaeneoic acid (EPA) and docosahexaenoic acid (DHA) have a potential mode of action through incorporation into immune cell membranes and alteration of the patterns of eicosanoid production, and anti-inflammatory molecules such as resolvins, protectins and maresins [33]. This leads to a down-regulation in inflammatory cytokine production (e.g. IL-1, IL-6, TNFα) along with other cell mediators.

n-3 PUFA supplementation has been investigated in dialysis patients. A recent systematic review and meta-analysis of RCT studies was performed [34]. Specifically for inflammation, 20 studies were assessed, and they found that overall n-3 PUFA have positive effects on reducing markers of inflammation (CRP, IL-6 and TNF-a). Note also in their analysis of studies they found beneficial effects on haemoglobin and there were few adverse effects apart from gastrointestinal disturbances. A further recent meta-analysis of 11 RCT studies investigating the effects of n-3 PUFA in haemodialysis patients showed that supplementation may reduce markers of inflammation [35]. Analysis of studies showed that CRP levels significantly reduced in patients, and this was more pronounced in those patients with CRP ≥5 mg/dl. The range of doses in the study were from 1.3 g/day to 3.0 g/day. However, levels of IL-6 and TNFα were not significantly altered, however only a small number of studies were assessed (four for IL-6 and three for TNFα). Other key points noted from both systematic reviews were that studies varied considerably in dosage used and duration of intervention.

n-3 PUFA may also have beneficial effects directly on skeletal muscle, and this is being investigated in a range of studies and models, e.g. cell-based, animal and human [36]. The current theories are that not only would n-3 PUFA supplementation down-regulate inflammatory cytokine production known to stimulate muscle proteolysis and insulin resistance, but n-3 PUFA may be incorporated into skeletal muscle cell membranes and potentially alter muscle protein balance. Activation of mammalian target of Rapamycin (mTOR) muscle protein synthetic pathways, enhancement of insulin sensitivity and mitochondrial function, and dampening of NF-κB signalling are possible effects [33,36]. A systematic review and meta-analysis of human studies was performed by Bird *et al.* in which they analysed 123 human studies (66 studies included in meta-analysis) in healthy and diseased population groups (e.g. cancer, COPD and dialysis patients), specifically investigating effects of n-3 PUFA on sarcopenia, lean body mass, skeletal muscle mass and muscle strength [37]. They found overall that n-3 PUFA supplementation may have a positive effect on lean body mass, skeletal muscle mass, and some measures of strength.

A recent randomized clinical trial study investigated using a specific fish oil-based intradialytic parenteral nutrition (IDPM) feed in HD patients (*n* = 38 patients with 18 in IDPN group) with PEW, for a 3-month period (38). Serum albumin increased (*P* = 0.01), as did spontaneous dietary intake (*P* = 0.04) and body weight (*P* = 0.01). Patient's malnutrition inflammation scores also significantly improved (*P* = 0.01). The inclusion criteria were that patients had to be considered to be suffering from PEW and unable to tolerate ONS. They reported no changes in markers of inflammation but commented on that this may be due to the lower dose of EPA delivered (∼2820 mg/week). As noted above it appears that probably in the region of 2–3 g/day of fish oil is necessary to achieve an effect on inflammatory markers.

## CLOSING COMMENTS

Due to the multifactorial causal nature of cachexia in kidney disease it seems most appropriate that a multimodal approach to treating this condition is necessary. Note that many of the exercise studies mentioned did not yield significant improvements to muscle mass, and this may be due to the catabolic nature of the condition (e.g. increased inflammation, heightened proteolysis and hypermetabolism), and hence requirement to simultaneously provide adequate/in excess energy and protein, and use strategies to reduce inflammation (e.g. omega-3 rich fish oils) and/or promote anabolism (e.g. use of pharmacological anabolic agents such as ghrelin mimetics or selective androgen receptor modulators (SARMS).

A recent paper highlighted above using a fish oil-based IDPN intervention [38]. This was commented on in terms of delivery of omega-3 PUFA, but it must be mentioned that for some patients IDPN might be essential for the delivery of energy and protein nutrition, and meeting requirements, especially in those cachectic patients who have difficulty with consuming ONS and have overall poor dietary compliance. IDPN is quite a novel and specialized area of renal nutrition and something that has been discussed in a recent review article, and readers are encouraged to refer this paper for interest [7].

One final issue to consider is the conflicting views around protein intake for the older CKD patient. Many patients with CKD tend to be relatively older, and in our renal cachexia study we found this to be the case (mean age: 67.6 years) [10]. Older patients will have greater chances of having comorbidities, sarcopenia and frailty, and a higher protein intake has been suggested for older people [39]. However, for the CKD population not on dialysis a lower protein has been suggested as in KDOQI guidelines. What this means for the older CKD patient at risk of malnutrition, and not yet on dialysis has been poorly studied, and has been subject of a recent Editorial discussion [40].

## CONCLUSION

There is good evidence that exercise and nutrition interventions for dialysis patients may have positive effects on physical strength/function and nutritional status. Future studies are urgently needed to assess specific combined multimodal treatment plans for patients with renal cachexia. Furthermore, to optimize the undertaking and adherence of these nutrition and exercise interventions they will need to be tailored and personalized to the individual patient.

## Acknowledgements

*We would like to acknowledge the team of international experts and researchers who have been involved with this work and are co-authors in recent paper published on this topic*[1^▪^].

### Financial support and sponsorship

*None.*

### Conflicts of interest

*There are no conflicts of interest.*

## REFERENCES AND RECOMMENDED READING

Papers of particular interest, published within the annual period of review, have been highlighted as:

▪ of special interest
▪▪ of outstanding interest
